# Design and Fabrication of a Microfluidic Viscometer Based on Electrofluidic Circuits

**DOI:** 10.3390/mi9080375

**Published:** 2018-07-27

**Authors:** Bo-Bi Tzeng, Yung-Shin Sun

**Affiliations:** Department of Physics, Fu-Jen Catholic University, New Taipei City 24205, Taiwan; 403166019@mail.fju.edu.tw

**Keywords:** viscometer, microfluidics, electrofluidic circuit

## Abstract

This paper reports a microfluidic viscometer based on electrofluidic circuits for measuring viscosities of liquid samples. The developed micro-device consists of a polydimethylsiloxane (PDMS) layer for electrofluidic circuits, a thin PDMS membrane, another PDMS layer for sample pretreatment, and a glass substrate. As the sample flows inside the microfluidic channel, its viscosity causes flow resistance and a pressure drop along this channel. This pressure drop, in turn, generates a hydraulic pressure which deforms the PDMS membrane, causing changes in the cross-sectional area and the electrical resistance of the electrofluidic resistor. This small resistance change is then measured via the electrofluidic Wheatstone bridge to relate the measured voltage difference to the fluidic viscosity. The performance of this viscometer was first tested by flowing nitrogen gas with controllable pressures into the device. The relationship between measured voltage difference and input gas pressure was analyzed to be linear in the pressure range of 0–15 psi. Another test using pure water indicated good linearity between measured voltage difference and flow rate in the rate range of 20–100 μL/min. Viscosities of glycerol/water solutions with volume/volume (*v*/*v*) concentrations ranging from 0 to 30% were measured, and these values were close to those obtained using commercially available viscometers. In addition, the sample-pretreatment layer can be used to mix and/or dilute liquid samples to desired concentrations. Therefore, this microfluidic device has potential for measurements of fluidic viscosity in a fast, accurate, and high-throughput manner.

## 1. Introduction

Viscosity is an important fluidic property that occurs between two surfaces of that fluid moving at different velocities. It is simply the frictional force among the molecules of that fluid. Measurements of the viscosity of biological samples such as blood, tissue fluids, and urine can be applied to disease diagnosis and prognosis. For example, estimates of urine viscosity provide a better characterization of the biological system and should result in more accurate modeling of bladder hyperthermia treatments [1]. The viscosity of urine, a Newtonian fluid [1,2,3], was shown to depend on the temperature and urinary constituents, and its value increased with the appearance proteinuria [1]. Maple syrup urine disease (MSUD), a metabolic disorder affecting the process of certain amino acids, is characterized by low-viscosity urine [4]. Urine viscosity was also used to evaluate homeostasis in heart surgery patients in the early postoperative period [5].

Conventional viscometers such as U-tube viscometers, falling ball viscometers, vibrational viscometers, rotational viscometers, electromagnetically spinning sphere (EMS) viscometers, and Stabinger Viscometers^TM^ are routinely used in laboratories. A vibrational viscometer measures the damping of an oscillating resonator immersed in a fluid, and this damping increases with increasing fluidic viscosity [6]. The Viscolite from Hydramotion (York, UK) is a commercially available vibrational viscometer with a detection range of 0–10,000 cP and an accuracy of 0.1 cP. A rotational viscometer works by measuring the torque needed to turn an object in a fluid, and this torque is a function of the fluidic viscosity. The Alpha series rotational viscometers from Fungilab (Barcelona, Spain) have a detection range of 1–2,000,000 cP and an accuracy of 10 cP. In an EMS viscometer, a metal sphere revolves under the influence of an external magnetic field, and its rotational speed depends on the viscosity of the fluid it is immersed in [7,8]. The EMS-1000 from Kyoto Electronics Manufacturing (Kyoto, Japan) measures viscosities from 0.1 to 100,000 cP. The Stabinger Viscometer^TM^ (Anton Paar, Graz, Austria), a modification of the classic Couette-type rotational viscometer, determines kinematic viscosities with a wide measuring range. The Stabinger Viscomete^TM^ SVM^TM^ 3000 measures viscosities from 0.2 to 20,000 cP and is applied to measurements of various samples [9,10,11].

All abovementioned, conventional viscometers have a few drawbacks, including usually large sample consumption, inability for real-time measurements, and expensive instruments. For example, the minimum volumes required in Viscolite and Alpha series are 100 mL and 10 mL, respectively. As a result, a variety of microfluidics-based viscometers were developed to overcome these issues. Micro-fabricated devices composed of micro-channels and fluidic components provide a miniature platform where the consumption of samples is significantly reduced and the micro-environment (such as temperature, pH value, and chemical concentration) is well controlled. With advances in techniques of micro-fabrication from glass and silicon substrates to polydimethylsiloxane (PDMS) and polymethylmethacrylate (PMMA) materials, the cost and time for fabricating microfluidic chips are greatly reduced, leading to more diverse applications of these devices. Early in 2005, Srivastava and Burns reported a micro-fabricated, glass-made nanoliter capillary viscometer for quick, easy, and inexpensive measurements of fluidic viscosities [12]. Based on capillary pressure-driven flow inside microfluidic channels, viscosities ranging from 1 to 5 cP were measured [12]. In the following year, the same group fabricated a self-calibrating, micro-fabricated capillary viscometer for analyzing non-Newtonian fluids [13]. This glass-made device monitored the capillary pressure-driven movement of the fluid sample whose velocity and shear rate varied with time. Viscosities in the range of 1–600 cP were measured with shear rates varying from 5 to 1000 s^−1^ [13]. Zheng et al*.* developed a PDMS viscometer by utilizing the high solubility and permeability of air in PDMS to generate Poiseuille flow in the degassed PDMS microfluidic device [14]. The viscosity of the fluid was obtained by measuring the distance the sample traveled and its flow velocity in the PDMS channel. This microfluidic viscometer was able to measure viscosities of Newtonian fluids ranging from 1 to 80 cP [14]. Kang and Yang proposed a microfluidic viscometer equipped with a fluid temperature controller for measuring the viscosities of both Newtonian and non-Newtonian fluids [15]. Solomon and Vanapalli demonstrated the first high-throughput microfluidic viscometer for simultaneously measuring the viscosities of multiple samples [16]. Other microfluidic viscometers based on diffusion, surface tension, velocimetry, flow rate sensing, and pressure sensing were also reported [17]. The state-of-the-art techniques in designing and fabricating microfluidic rheometers and viscometers are detailed in References [18,19,20,21,22,23].

In this study, a microfluidic viscometer based on electrofluidic circuits was developed. This microfluidic device consists of a PDMS layer for electrofluidic circuits, a thin PDMS membrane, another PDMS layer for sample pretreatment, and a glass substrate. The ionic liquid-based electrofluidic circuit can monitor pressure changes by measuring the corresponding resistance changes in the circuit; the working principles are detailed in References [18,24,25,26]. The idea of turning this pressure sensor into a viscometer is as follows: inside a microfluidic channel, the flow resistance increases with increasing fluidic viscosity, and this increasing flow resistance, in turn, increases the pressure drop along this channel. This pressure drop generates a hydraulic pressure at the pressure-sensing area, causing changes in the cross-sectional area and the electrical resistance of the electrofluidic circuit. This resistance change is then measured by means of the electrofluidic Wheatstone bridge. The present microfluidic viscometer offers some advantages as compared to that shown in the article published earlier this year [18]. Firstly, the sample-pretreatment layer can be used to mix and/or dilute liquid samples. By redesigning this device to have multiple samples of different concentrations [27,28,29], with each corresponding to one electrofluidic sensing region, it can serve as a platform for measurements of fluidic viscosity in a fast, accurate, and high-throughput manner. Secondly, cells can be cultured inside this microfluidic chip for monitoring their responses to fluidic shear stress and viscosity. Thirdly, this microfluidic viscometer is capable of measuring low-viscosity liquids: a glycerol solution of 2.5% (*v*/*v* in water) was measured to have a viscosity of 0.94 cP (the viscosity of water is around 0.89 cP at 25 °C). 

## 2. Materials and Methods 

### 2.1. Design and Fabrication of the Microfluidic Chip

**Figure 1:**
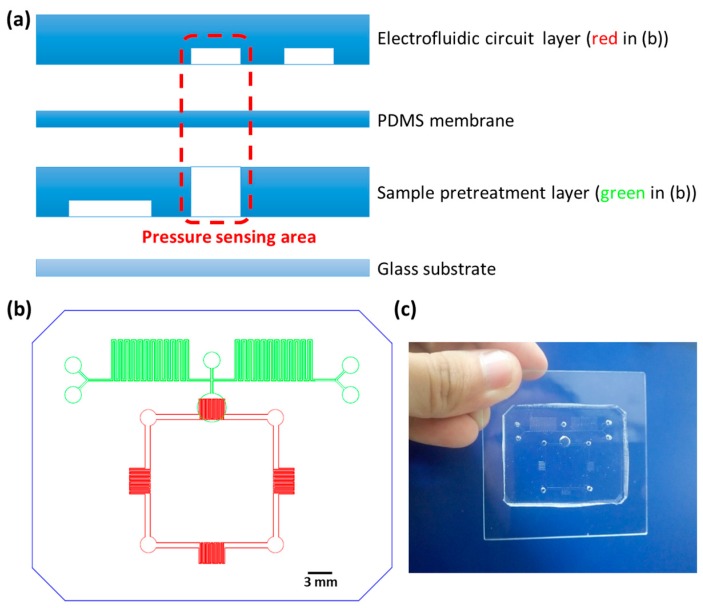
(**a**) The layer-by-layer scheme of the microfluidic chip (side view). (**b**) The sample-pretreatment layer (green) and the electrofluidic circuit layer (red) of the microfluidic chip (top view). (**c**) A picture of the microfluidic chip. PDMS—polydimethylsiloxane.

The layer-by-layer scheme of the microfluidic chip is shown in Figure 1a. The topmost layer had four electrofluidic resistors which constructed the Wheatstone bridge for pressure sensing (see the red part in Figure 1b). The third layer had two symmetric parts serving for sample pretreatment (see the green part in Figure 1b), with each part consisting of two inlets and a continuous zigzag microchannel for mixing and diluting liquid samples. This zigzag structure is very commonly used in microfluidics for mixing and diluting purposes [27,28,29,30,31,32]. Compared to a straight microchannel, the total length of mixing is significantly increased in a zigzag microchannel. Moreover, by hitting the wall frequently, the mixing process is more efficient in a zigzag microchannel. These two layers were fabricated using the standard soft-lithography technique including photoresist SU-8 (2025, MicroChem, Westborough, MA, USA) molding and elastomeric material PDMS (Sylgard 184, Dow Corning, Midland, MI, USA) curing. The heights and widths of the fluidic channels (in both the electrofluidic circuit layer and the fluidic layer), verified by α-step (Dektak XT, Bruker, Billerica, MI, USA), were about 50 and 200 μm, respectively. As illustrated in Figure 1b, on the sample pretreatment layer, a pressure transduction hole (diameter = 3.5 mm) was punched through and aligned with one of the four resistors on the electrofluidic circuit layer. The PDMS membrane with a thickness of 100 μm was fabricated by spinning a PDMS precursor (1:10 *w*/*w* of curing agent to base) onto a silicon wafer at 750 rpm for 40 s, baking the membrane-coated wafer inside a vacuum oven at 60 °C for 3 h, and peeling the membrane off the wafer. The electrofluidic circuit layer was punched using a 1.5-mm-diameter biopsy punch for ionic liquid injection and resistance measurement (see the four red circles in Figure 1b). It was permanently bonded to the PDMS membrane via oxygen plasma surface treatment at 18 W for 40 s under an O_2_ pressure of 600 mTorr (PDC-32G, Harrick Plasma, Ithaca, NY, USA). The sample-pretreatment layer, punched for gas/liquid sample injection (see the five green circles in Figure 1b), was then bonded to the bottom of the PDMS membrane via the same plasma treatment. Finally, the whole assembly was oxygen plasma-boned to a 5 cm × 5 cm glass substrate. A picture of the integrated microfluidic device is shown in Figure 1c. 

### 2.2. Theoretical Derivation and Simulation

The working principles of the pressure-sensing scheme in this device are detailed in References [18,24,25,26]. Inside a microfluidic channel, the fluidic viscosity (*μ*) is proportional to the flow resistance (*R_flow_*), this resistance is proportional to the pressure drop along this channel (Δ*P*), and this pressure drop generates a hydraulic pressure (*P_H_*) at the pressure-sensing area, causing changes in the cross-sectional area (Δ*A*) and the electrical resistance (Δ*R*) of the electrofluidic circuit. The basic idea can be expressed as

(1) $$\;\Delta P\propto P_{H}\propto \Delta A\propto \Delta R\propto \Delta V_{M}\;\mathrm{and}\;\mu \propto R_{flow}\propto \frac{d\Delta P}{dQ}\propto \frac{d\Delta V_{M}}{dQ},\;$$

where Δ*V_M_* is the measured voltage difference in the electrofluidic Wheatstone bridge and *Q* is the flow rate of the liquid sample.

The resistance (*R*) of each electrofluidic resistor is related to the resistivity (*ρ*) of the ionic liquid, the cross-sectional area (*A*) of the microfluidic channel, and the length (*l*) of that channel as [25]

(2) $$\;R=\rho \frac{l}{A}\;$$

**Figure 2:**
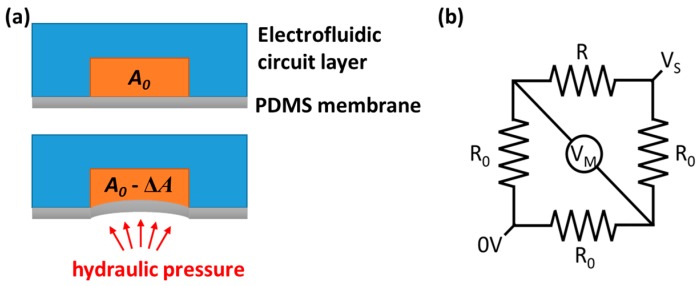
(**a**) The hydraulic pressure deforms the PDMS membrane. (**b**) The electrofluidic Wheatstone bridge.

As shown in Figure 2a, when the liquid sample passes through the pressure transduction hole, the hydraulic pressure deforms the PDMS membrane and changes the cross-section of the electrofluidic resistor by Δ*A*. This, in turn, alters the resistance of the electrofluidic resistor by Δ*R*, expressed as [25,28,33]

(3) $$\;∆R\approx \left(\frac{\rho l}{A_{0}^{2}}\right)\cdot ∆A,\;$$

where *A_0_* is the cross-section of the undeformed electrofluidic resistor.

This small resistance change is measured using the Wheatstone bridge built in the electrofluidic circuit layer. In Figure 2b, the measured voltage (*V_M_*) is proportional to the applied voltage (*V_s_*) as [25,28]

(4) $$\;V_{M}=\left(\frac{R_{0}}{R_{0}+R_{0}}-\frac{R_{0}}{R+R_{0}}\right)\cdot V_{S}=\left(\frac{1}{2}-\frac{R_{0}}{R+R_{0}}\right)\cdot V_{S},\;$$

where *R_0_* is the resistance of the undeformed electrofluidic resistor.

As *R* is replaced with *R_0_* + Δ*R*, the measured voltage difference (Δ*V_M_*) can be expressed as [25,28]

(5) $$\;∆V_{M}=\left[\frac{1}{2}-\frac{R_{0}}{\left(R_{0}+∆R\right)+R_{0}}\right]\cdot V_{S}=\left(\frac{\frac{1}{2}\cdot ∆R}{2R_{0}+∆R}\right)\cdot V_{S}.\;$$

For Δ*R* << *R_0_* and from Equations (3) and (5), the relationship between Δ*V_M_* and Δ*A* can be expressed as [25]

(6) $$\;∆V_{M}\approx \left(\frac{V_{S}}{4R_{0}}\right)\cdot ∆R\approx \left(\frac{V_{S}}{4R_{0}}\right)\cdot \left(\frac{\rho l}{A_{0}^{2}}\right)\cdot ∆A.\;$$

It follows that the hydraulic pressure (*P_H_*), and thus, the pressure drop (Δ*P*) are proportional to the measured voltage difference (Δ*V_M_*). Next, the flow resistance (*R_flow_*) is proportional to the pressure drop along the microfluidic channel (Δ*P*), the flow rate of the liquid sample (*Q*), the fluidic viscosity (*μ*), and the dimension of the channel (*w*: width; *h*: height; *L*: length) as [25]

(7) $$\;R_{flow}=\frac{d∆P}{dQ}=\frac{12\mu L}{wh^{3}}{\left\{1-\frac{h}{w}\left[\frac{192}{\pi^{5}}\overset{\infty }{\underset{n=1}{\sum }}\frac{1}{n^{5}}\tanh\left(\frac{n\pi w}{h}\right)\right]\right\}}^{-1}.\;$$

The viscosity is proportional to the slope in the Δ*P*–*Q* curve, and from Equation (6), it is also proportional to the slope in the Δ*V_M_*–*Q* curve. 

Equation (7) is used to calculate the pressure drop by assigning a finite summation up to *n* = *N*, *w* = 200 μm, *h* = 50 μm, *L* = 13.2 cm, *μ* = 0.89 cP for water at 25 °C, and *Q* = 100 μL/min. The calculation was performed using the Fortran programming language (version 95). Moreover, the same parameters were used in the COMSOL Multiphysics software (version 4.4, COMSOL, Burlington, MA, USA) for simulations on the pressure drop (via the “Laminar Flow” module) and the sample mixing process (via the “Transport of Dilute Species” module). The purposes of performing the COMSOL simulation are to show that (1) the calculation in Equation (7) is correct, and (2) the sample-pretreatment layer can effectively mix two samples of different concentrations.

### 2.3. Experimental Setup and Procedure

Four 1-mL plastic syringes with 16-gauge flat needles (Terumo, Somerset, NJ, USA) were filled with ionic liquid, 1-ethyl-3-methylimidazolium dicyanamide (370865-89-7, Alfa Aesar, Ward Hill, MA, USA), and inserted into the four holes in the electrofluidic circuit layer. The liquid was gently pushed into the electrofluidic circuit to avoid bubble formation. Electrical wires were wound around the needles and connected to a data acquisition board (NI-DAQ USB-6009, National Instruments, Austin, TX, USA) for applying and receiving electrical signals through the customized LABVIEW program (version 2011, National Instruments, Austin, TX, USA). This program was also written to control the pressure controller (ALI-PCD-15PSIG-D, Alicat Scientific, Tucson, AZ, USA) and syringe pumps (NE-1000, New Era, Farmingdale, NY, USA) for varying the pressure of gas and the flow rate of liquid samples, respectively. To test the pressure-sensing performance of this microfluidic device, nitrogen gas was flowed into the sample pretreatment layer via one sample injection hole (with other four holes sealed). A pressure increment of 0.5 psi was applied using the pressure controller to monitor the corresponding changes in measured voltage difference. To conduct experiments on liquids, samples (pure water or glycerol/water solutions) were flowed into the sample-pretreatment layer via two syringes and two sample-injection holes on the same side (with one hole on the other side serving as the outlet and the other two holes sealed). The flow rates inside the microfluidic channel were changed from 20 to 100 μL/min with an increment of 20 μL/min and a total waiting time of 10 min to monitor the corresponding changes in measured voltage difference.

## 3. Results

### 3.1. Calculation and Simulation

**Figure 3:**
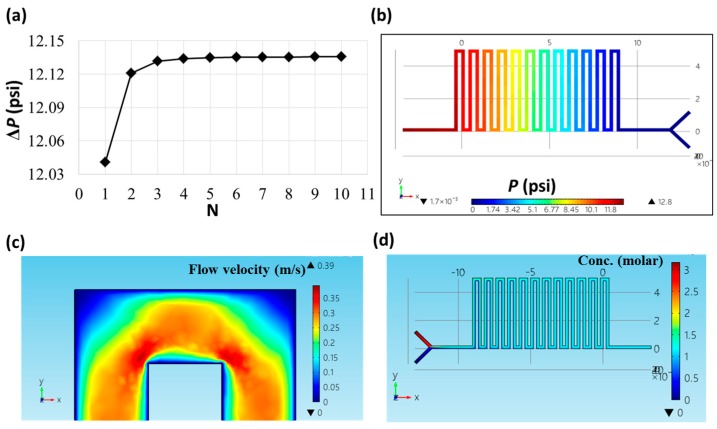
(**a**) Calculation of the pressure drop using the Fortran language. (**b**) The COMSOL simulation of the hydraulic pressure inside the microfluidic channel. (**c**) The COMSOL simulation of the flow velocity around one corner of the microfluidic channel. (**d**) The COMSOL simulation of the mixing process in the sample-pretreatment layer.

The pressure drop between the inlets and the pressure transduction hole was calculated using the Fortran language. In Equation (7), for values of *N* larger than 5, Δ*P* was constant and close to 12.135 psi under a flow rate of 100 μL/min, as shown in Figure 3a. In the COMSOL simulation indicated in Figure 3b, the pressure drop was around 12.8 psi (the difference in hydraulic pressures between the leftmost, dark-red area and the right-most, blue area). This value was slightly (~5%) higher than that calculated using Fortran. Figure 3c shows the flow velocity around one corner of the microfluidic channel. Liquids tended to flow along the inner corner of the channel, leading to an increase in flow resistance and pressure drop compared with the assumed straight channel in Equation (7). The sample mixing process was also simulated using the COMSOL software. In Figure 3d, by flowing two solutions with concentrations of 0 and 3 M into the two inlets at the same flow rates, a uniform solution with a concentration of about 1.5 M was obtained in the outlet.

### 3.2. Gas and Pure Water Test

**Figure 4:**
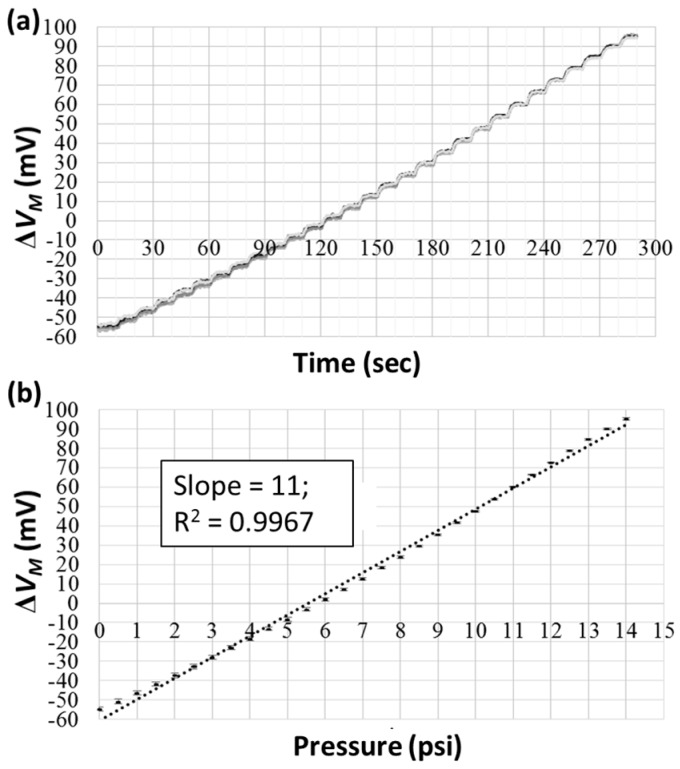
The pressure-sensing performance of the device verified using the nitrogen gas sample. (**a**) The measured voltage differences from five successive measurements. (**b**) The measured voltage differences plotted against gas pressures.

To investigate the pressure-sensing performance, nitrogen gas was introduced into the microfluidic channel to deform the PDMS membrane. This gas was supplied from an N_2_ gas cylinder and regulated via a pressure controller. The gas pressure was controlled to increase from 0 to 14 psi at an increment of 0.5 psi, and the waiting time between two pressure values was 10 s, much longer than the responding time of the pressure controller. The measured voltage differences from five successive measurements are shown in Figure 4a, indicating linear responses and good repeatability. The average values with standard deviations (over the last 5 s for each gas pressure and over five measurements) were plotted against the gas pressure, as shown in Figure 4b. An offset voltage difference of around −60 mV was recorded at zero pressure. From linear fitting, a slope of 11 mV/psi indicated that a pressure of 1 psi will lead to an 11-mV measured voltage difference. Moreover, the good linearity (*R*^2^ = 0.9967) verified the applicability of the present device for pressure and viscosity measurements. By inserting Δ*V_M_* = 152.6 mV and *V_S_* = 5 V into Equation (6), the resistance change Δ*R* under a gas pressure of 14 psi was about 12.2% of the original value.

**Figure 5:**
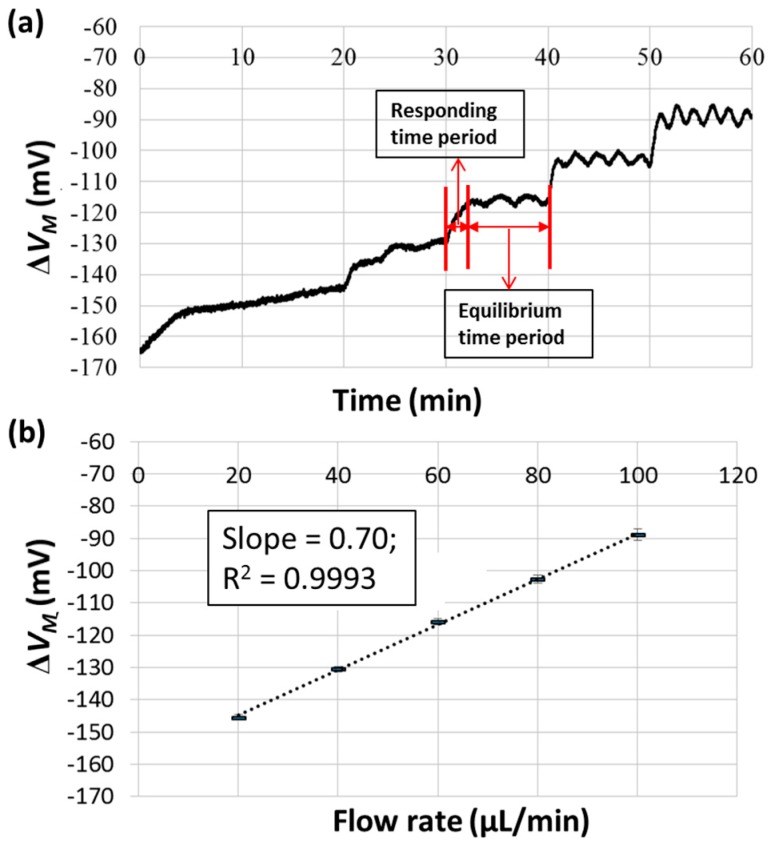
The pressure-sensing performance of the device verified using the pure water sample. (**a**) The measured voltage differences versus time. (**b**) The measured voltage differences plotted against liquid flow rate.

The performance of this device in liquid pressure measurements was studied by flowing pure water into the microfluidic channel to deform the PDMS membrane. The flow rate of the water was controlled via syringe pumps and increased from 10 to 50 μL/min with an increment of 10 μL/min and a total waiting time of 10 min. Since there were two inlets, this corresponded to flow rates of 20–100 μL/min with an increment of 20 μL/min inside the microfluidic channel. The outlet of the microfluidic channel was exposed to the atmosphere, so the measured voltage difference was related to the pressure drop along the channel and the hydraulic pressure at the pressure transduction hole. Figure 5a shows the corresponding Δ*V_M_* plotted against time. As indicated, the responding time was about 3 min; thus, the data from the last 5 min were taken for the purpose of statistics. Ripples in the voltage were observed, probably due to the stepper motor of the pump used to push the syringe. The average values of Δ*V_M_* with standard deviations (over three successive measurements) were plotted against the flow rate, as shown in Figure 5b. A slope of 0.70 mV·min/μL and an *R*^2^ of 0.9993 were obtained from linear fitting, indicating the good linearity in measuring liquid samples over this flow rate range. Therefore, by assigning the viscosity of pure water (*μ* = 0.89 cP at 25 °C) to its slope (*d*Δ*V_M_*/*dQ* = 0.70 mV·min/μL, which is proportional to *d*Δ*P*/*dQ* in Equation (7)), the viscosities of other liquids can be obtained from their slopes of measured voltage difference versus flow rate.

### 3.3. Samples with Different Viscosities

**Figure 6:**
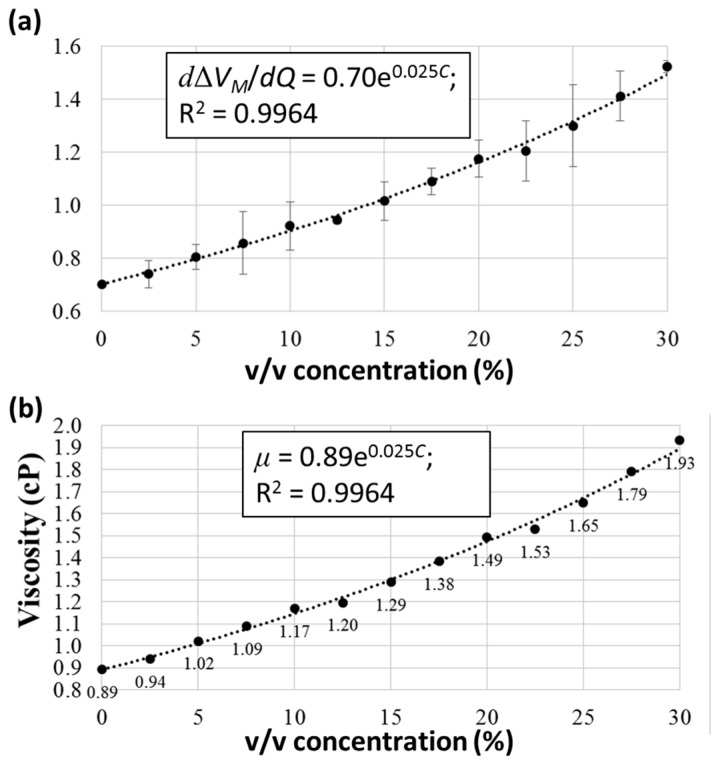
Viscosity measurements of glycerol/water solutions. (**a**) The slopes *d*Δ*V_M_*/*dQ* at different concentrations. (**b**) The viscosities at different concentrations.

Glycerol solutions of different concentrations were obtained by flowing pure water and 30% *v*/*v* glycerol solution in water into the two inlets of the microfluidic channel at desired flow rates. For example, with a desired flow rate of 20 μL/min inside the channel, a concentration of 15% was achieved by injecting water and 30% glycerol solution at flow rates of 10 and 10 μL/min, respectively. A concentration of 2.5% was achieved by injecting water and 30% glycerol solution at flow rates of 18.3 and 1.7 μL/min, respectively. Thirteen samples, with concentrations ranging from 0% to 30% at an increment of 2.5%, were measured under flow rates of 20–100 μL/min and an increment of 20 μL/min inside the microfluidic channel. The average values of *d*Δ*V_M_*/*dQ* with standard deviations (over three independent measurements) were plotted against the concentration, as shown in Figure 6a. Furthermore, by assigning a viscosity of 0.89 cP to 0% concentration (pure water), the viscosities at different concentrations could be obtained.

As clearly observed in Figure 6b, the linearity obtained through linear regression to these points was not very good (*R*^2^ = 0.9804, data not shown). According to the literature, the relationship between the viscosity of a glycerol/water solution and its concentration is better described by an exponential curve [34]. The relationship among the viscosities of pure water (*μ_w_*), pure glycerol (*μ_g_*), and glycerol/water mixtures (*μ*) can be expressed as

(8) $$\;\mu =\mu_{w}^{\alpha }\mu_{g}^{1-\alpha },\;$$

where *α* is the weighting factor between 0 and 1. By letting $A=\ln\left(\frac{\mu_{w}}{\mu_{g}}\right)$, Equation (8) can be rewritten as

(9) $$\;\mu =\mu_{g}e^{\left(A\alpha \right)}.\;$$

Another parameter *β* is defined to relate the weighting factor to the concentration of the glycerol/water (*C_m_*) solution as

(10) $$\;\beta =\alpha -1+C_{m}.\;$$

For small values of *C_m_*, *β* depends linearly on *C_m_*

(11) $$\;\beta =aC_{m}\;$$

The parameter *a* was experimentally determined to be *a* = 0.705 − 0.0017*T*, where *T* is the temperature in °C [34]. By inserting Equation (11) into Equation (10), the weighting factor can be expressed as

(12) $$\;\alpha =1+\left(a-1\right)C_{m}.\;$$

Then, Equation (9) can be rewritten as

(13) $$\;\mu =\mu_{g}e^{\left(A+A\left(a-1\right)C_{m}\right)}=\mu_{w}e^{\left(A\left(a-1\right)C_{m}\right)}.\;$$

Given that at 25 °C, $A=\ln\left(\frac{\mu_{w}}{\mu_{g}}\right)=\ln\left(\frac{0.89}{905.68}\right)=-6.925$ and *a* = 0.705 − 0.0017 × 25 = 0.6625, the viscosity of the glycerol/water solution can be expressed as

(14) $$\;\mu =\mu_{w}e^{\left(2.34C_{m}\right)}.\;$$

By inserting *μ_w_* = 0.89 into and changing *C_m_* to *v*/*v* percentage in Equation (14), the viscosity is related to the concentration as

(15) $$\;\mu =0.89e^{\left(0.0234C_{m}\right)}.\;$$

This equation, with an experimental variable, was used to fit the points in Figure 6b. As shown, an *R*^2^ of 0.9964 indicated a good fitting, and the experimental variable 0.025 was 6.83% higher than the calculated value 0.0234 in Equation (15). This could possibly be due to the deformation of PDMS channels during experiments [33,35].

### 3.4. Comparison with Other Devices

**Figure 7:**
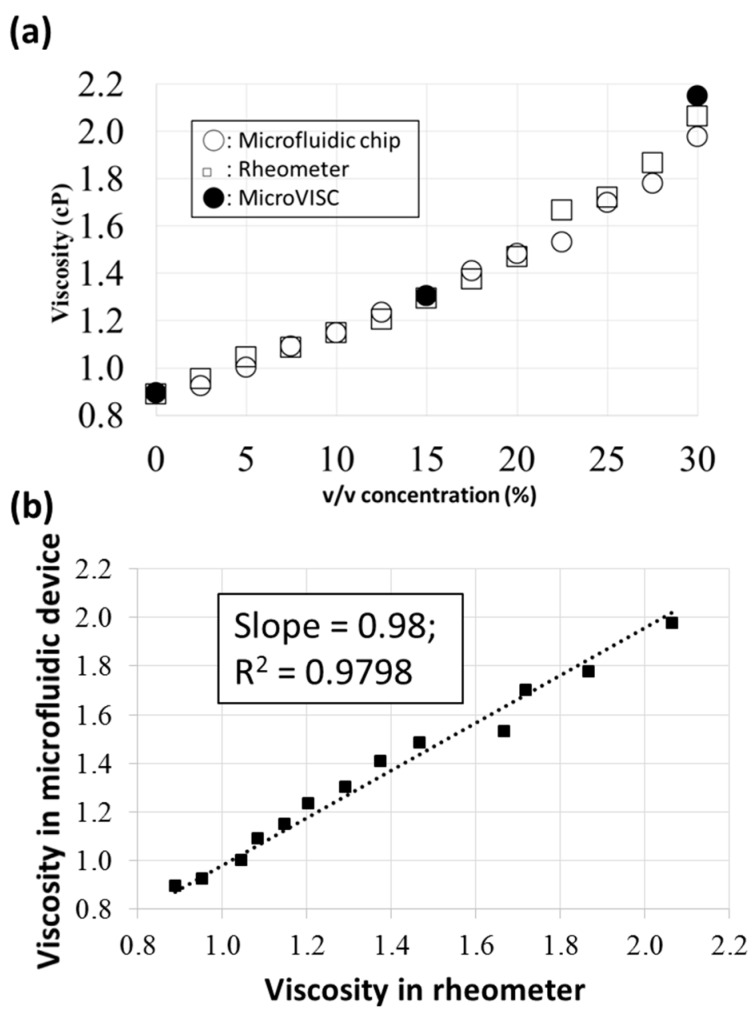
(**a**) Comparison among viscosities obtained from the present microfluidic device and two commercially available viscometers. (**b**) The viscosities measured by the rheometer versus those measured by the microfluidic device.

The viscosities obtained in the present microfluidic chip were compared to those determined from two commercially available devices, a viscometer (microVISC, RheoSense, San Ramon, CA, USA) and a rheometer (AR2000ex, TA Instruments, New Castle, DE, USA). As shown in Figure 7a, the differences overall were small. For example, compared with the rheometer, the differences were 0.001 cP (0.1%), 0.089 cP (4.5%), and 0.137 cP (8.9%) at concentrations of 10%, 30%, and 22.5%, respectively. Moreover, compared with the viscometer, the differences were 0.001 cP (0.1%) and 0.174 cP (8.8%) at concentrations of 15% and 30%, respectively. Also, Figure 7b shows the viscosities measured by the AR2000ex rheometer versus those measured by the microfluidic device. A fitted slope of 0.98 again indicates small measured differences. These results suggest that the present microfluidic device can serve as an alternative to other commercialized viscometers.

## 4. Conclusions 

This paper successfully demonstrated a microfluidic viscometer based on electrofluidic circuits. Constructed using elastomeric PDMS, this micro-device provides several advantages over traditional viscometers, including portability, disposability, effective cost, simple fabrication, and low sample consumption. Inside the microfluidic channel, the fluidic viscosity is proportional to the flow resistance and the pressure drop along this channel. This pressure drop generates a hydraulic pressure at the transduction hole, causing changes in the cross-sectional area and the electrical resistance of the electrofluidic circuit. This resistance change is, in turn, measured via the electrofluidic Wheatstone bridge. By combining the electrofluidic circuit layer with another sample-pretreatment layer, this micro-viscometer offers an additional benefit of mixing and/or diluting liquid samples. Viscosities of glycerol/water solutions with *v*/*v* concentrations ranging from 0 to 30% were measured, and these values were close to those obtained using commercially available devices. In the near future, a microfluidic viscometer based on the concepts of the present device will be designed and fabricated to (1) measure viscosities of biological samples in a fast, accurate, and high-throughput manner, and (2) culture cells for monitoring their responses to fluidic shear stress and viscosity.

## References

[1] Inman B.A., Etienne W., Rubin R., Owusu R.A., Oliveira T.R., Rodriques D.B., Maccarini P.F., Stauffer P.R., Mashal A., Dewhirst M.W. (2013). The impact of temperature and urinary constituents on urine viscosity and its relevance to bladder hyperthermia treatment. Int. J. Hyperth..

[2] Gomez-Blanco J.C., Martinez-Reina F.J., Cruz D., Pagador J.B., Sanchez-Margallo F.M., Soria F. (2016). Fluid Structural Analysis of Urine Flow in a Stented Ureter. Comput. Math. Method. Med..

[3] Kim K.W., Choi Y.H., Lee S.B., Baba Y., Kim H.H., Suh S.H. (2017). Analysis of Urine Flow in Three Different Ureter Models. Comput. Math. Method. Med..

[4] Maple Syrup Urine Disease (MSUD). http://ashlandscience.shoutwiki.com/wiki/Maple_Syrup_Urine_Disease_(MSUD).

[5] Roitman E.V., Dement’eva I.I., Kolpakov P.E. (1995). [Urine viscosity in the evaluation of homeostasis in heart surgery patients in the early postoperative period]. Klin. Lab. Diagn..

[6] Yabuno H., Higashino K., Kuroda M., Yamamoto Y. (2014). Self-excited vibrational viscometer for high-viscosity sensing. J. Appl. Phys..

[7] Fukunaga K., Onuki M., Ohtsuka Y., Hirano T., Sakai K., Ohgoe Y., Katoh A., Yaguchi T., Funakubo A., Fukui Y. (2013). Blood viscometer applying electromagnetically spinning method. J. Artif. Organs.

[8] Sakai K., Hirano T., Hosoda M. (2010). Electromagnetically Spinning Sphere Viscometer. Appl. Phys. Express.

[9] Almasi M. (2015). Temperature dependence and chain length effect on density and viscosity of binary mixtures of nitrobenzene and 2-alcohols. J. Mol. Liq..

[10] Torin-Ollarves G.A., Martin M.C., Chamorro C.R., Segovia J.J. (2014). Densities, viscosities, and isobaric heat capacities of the system (1-butanol + cyclohexane) at high pressures. J. Chem. Thermodyn..

[11] Regueira T., Lugo L., Fandino O., Lopez E.R., Fernandez J. (2011). Compressibilities and viscosities of reference and vegetable oils for their use as hydraulic fluids and lubricants. Green Chem..

[12] Srivastava N., Davenport R.D., Burns M.A. (2005). Nanoliter viscometer for analyzing blood plasma and other liquid samples. Anal. Chem..

[13] Srivastava N., Burns M.A. (2006). Analysis of non-Newtonian liquids using a microfluidic capillary viscometer. Anal. Chem..

[14] Han Z., Tang X., Zheng B. (2007). A PDMS viscometer for microliter Newtonian fluid. J. Micromech. Microeng..

[15] Kang Y.J., Yang S. (2013). Integrated microfluidic viscometer equipped with fluid temperature controller for measurement of viscosity in complex fluids. Microfluid. Nanofluid..

[16] Solomon D.E., Vanapalli S.A. (2014). Multiplexed microfluidic viscometer for high-throughput complex fluid rheology. Microfluid. Nanofluid..

[17] Gupta S., Wang W.S., Vanapalli S.A. (2016). Microfluidic viscometers for shear rheology of complex fluids and biofluids. Biomicrofluidics.

[18] Lee T.A., Liao W.H., Wu Y.F., Chen Y.L., Tung Y.C. (2018). Electrofluidic Circuit-Based Microfluidic Viscometer for Analysis of Newtonian and Non-Newtonian Liquids under Different Temperatures. Anal. Chem..

[19] Arosio P., Hu K., Aprile F.A., Muller T., Knowles T.P. (2016). Microfluidic Diffusion Viscometer for Rapid Analysis of Complex Solutions. Anal. Chem..

[20] Zou M., Cai S., Zhao Z., Chen L., Zhao Y., Fan X., Chen S. (2015). A novel polydimethylsiloxane microfluidic viscometer fabricated using microwire-molding. Rev. Sci. Instrum..

[21] DeLaMarre M.F., Keyzer A., Shippy S.A. (2015). Development of a simple droplet-based microfluidic capillary viscometer for low-viscosity Newtonian fluids. Anal. Chem..

[22] Wang Z., Tan L., Pan X., Liu G., He Y., Jin W., Li M., Hu Y., Gu H. (2017). Self-Powered Viscosity and Pressure Sensing in Microfluidic Systems Based on the Piezoelectric Energy Harvesting of Flowing Droplets. ACS Appl. Mater. Interfaces.

[23] Li Y., Ward K.R., Burns M.A. (2017). Viscosity Measurements Using Microfluidic Droplet Length. Anal. Chem..

[24] Liu M.C., Shih H.C., Wu J.G., Weng T.W., Wu C.Y., Lu J.C., Tung Y.C. (2013). Electrofluidic pressure sensor embedded microfluidic device: A study of endothelial cells under hydrostatic pressure and shear stress combinations. Lab Chip.

[25] Wu C.Y., Liao W.H., Tung Y.C. (2011). Integrated ionic liquid-based electrofluidic circuits for pressure sensing within polydimethylsiloxane microfluidic systems. Lab Chip.

[26] Wu C.Y., Lu J.C., Liu M.C., Tung Y.C. (2012). Integrated electrofluidic circuits: pressure sensing with analog and digital operation functionalities for microfluidics. Lab Chip.

[27] Lo K.Y., Wu S.Y., Sun Y.S. (2016). A microfluidic device for studying the production of reactive oxygen species and the migration in lung cancer cells under single or coexisting chemical/electrical stimulation. Microfluid. Nanofluid..

[28] Lo K.Y., Zhu Y., Tsai H.F., Sun Y.S. (2013). Effects of shear stresses and antioxidant concentrations on the production of reactive oxygen species in lung cancer cells. Biomicrofluidics.

[29] Huang C.H., Hou H.S., Lo K.Y., Cheng J.Y., Sun Y.S. (2017). Use microfluidic chips to study the effects of ultraviolet lights on human fibroblasts. Microfluid. Nanofluid..

[30] Mengeaud V., Josserand J., Girault H.H. (2002). Mixing processes in a zigzag microchannel: Finite element simulations and optical study. Anal. Chem..

[31] Chen J.K., Yang R.J. (2007). Electroosmotic flow mixing in zigzag microchannels. Electrophoresis.

[32] Kim S., Kim H.J., Jeon N.L. (2010). Biological applications of microfluidic gradient devices. Integr. Biol..

[33] Hosokawa K., Hanada K., Maeda R. (2002). A polydimethylsiloxane (PDMS) deformable diffraction grating for monitoring of local pressure in microfluidic devices. J. Micromech. Microeng..

[34] Cheng N.S. (2008). Formula for the viscosity of a glycerol-water mixture. Ind. Eng. Chem. Res..

[35] Gervais T., El-Ali J., Gunther A., Jensen K.F. (2006). Flow-induced deformation of shallow microfluidic channels. Lab Chip.

